# Spotlight on Proteases: Roles in Ovarian Health and Disease

**DOI:** 10.3390/cells14120921

**Published:** 2025-06-18

**Authors:** Bhawna Kushawaha, Emanuele Pelosi

**Affiliations:** Department of Biochemistry and Molecular Biology, Indiana University, Indianapolis, IN 46202, USA; bkushaw@iu.edu

**Keywords:** proteases, ovary, matrix metalloproteinases, cathepsins, ovarian reserve, ovarian cancer, polycystic ovary syndrome, primary ovarian insufficiency

## Abstract

Proteases play crucial roles in ovarian folliculogenesis, regulating several processes from primordial follicle activation to ovulation and corpus luteum formation. This review synthesizes the current knowledge on the diverse functions of proteases in ovarian physiology and pathology. We discuss the classification and regulation of proteases, highlighting their importance in extracellular matrix remodeling, cell signaling, and apoptosis during ovarian follicular development. We explore the roles of several proteases including matrix metalloproteinases, tissue inhibitors of metalloproteinases, the plasminogen activator system, and cathepsins, and their roles in the critical functions of ovarian biology including follicle dynamics and senescence. Furthermore, we address the involvement of proteases in ovarian pathologies, including cancer, polycystic ovary syndrome, and primary ovarian insufficiency. By integrating recent findings from clinical genomics and animal models, this review provides a comprehensive overview of protease functions in the ovary, emphasizing their potential use for therapeutic interventions in reproductive medicine.

## 1. Introduction

**Figure 1:**
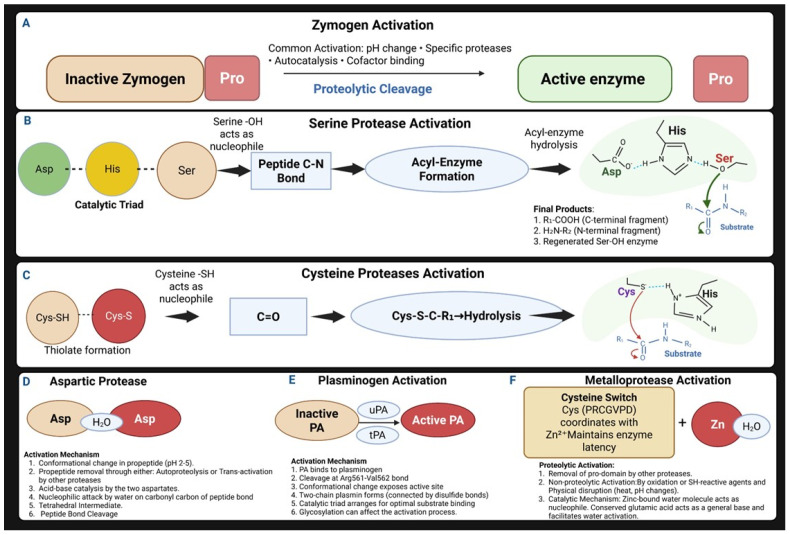
Mechanisms of protease activation: (**A**) zymogen activation through proteolytic cleavage triggered by factors including specific proteases, autocatalysis, or cofactor binding; (**B**) activation of serine proteases featuring the Asp–His–Ser catalytic triad, which facilitates nucleophilic attack on peptide bonds; (**C**) activation of cysteine proteases where the thiolate group serves as the nucleophile; (**D**) activation of aspartic proteases involving dual aspartate residues that use water for peptide bond hydrolysis; (**E**) mechanisms of activation of tissue–type (tPA) and urokinase–type (uPA) plasminogen activators; (**F**) activation of metalloproteases through the cysteine switch and zinc-mediated catalysis.

Several ovarian processes from follicle activation to the maturation and release of oocytes require the catalytic action of protease enzymes [1,2,3,4]. Since first reported in 1905 by P. A. Levene, proteases remain at the forefront of basic and clinical research due to their regulatory activity of fundamental and ubiquitous cellular functions [5]. Recent innovations in large-scale genome sequencing and high-throughput differential screening methods have established that the mouse and human genomes contain 628 and 566 protease-encoding genes, respectively [6,7,8,9,10]. In humans, there are 273 extracellular proteases, 277 intracellular proteases, and 16 integral membrane proteases. The mouse degradome includes at least 628 members with 514 true orthologs of human proteases [11,12,13]. The specific function of several proteases remains unclear, and roughly 100 of these proteins are believed to be inactive due to the lack of critical catalytic domains [14]. Proteases are classified into two main groups: exopeptidases, which cleave peptide bonds near the amino or carboxy terminus (aminopeptidases and carboxypeptidases), and endopeptidases, which cut inside proteins, away from the ends. Based on the type of catalytic domain, proteases are grouped into six classes [15,16,17]. Serine, cysteine, and threonine proteases use nucleophilic amino acids for cleavage, whereas aspartic, glutamic, and metalloproteases activate water molecules as nucleophiles for the hydrolysis of peptide bonds (Figure 1) [18,19,20]. Most proteases are synthesized as inactive precursors called zymogens or proenzymes. These contain inhibitory pro-domains that prevent premature proteolytic activity [17,21]. Proteases lacking pro-domains typically require cofactor binding or post-translational modifications to achieve an active state (Figure 1) [22,23,24].

Proteases modulate growth factor/cytokine bioavailability, control the levels of cell surface/receptor proteins, and are involved in the reorganization of the extracellular matrix (ECM) [25,26,27]. Therefore, their activity requires fine regulation through several mechanisms including inhibition by specific proteins, degradation by other proteases, and subcellular relocalization [28].

**Figure 2:**
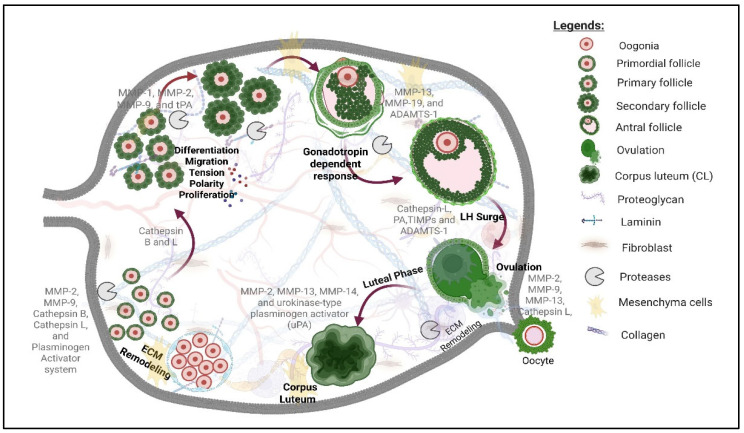
Schematic Overview: Ovarian Follicle Development Through Corpus Luteum Formation and Involvement of Various Protease. Proteases regulate critical processes including follicular activation, differentiation, migration, ECM remodeling, and gonadotropin-dependent responses. Several extracellular matrix components (proteoglycans, laminin, and collagen) and cell types (fibroblasts and mesenchymal cells) interact with follicle cells during the developmental process. Proteases are involved in ECM remodeling, which is critical for follicle growth, oocyte maturation, and luteal phase transition.

In the ovary, several proteases, including matrix metalloproteinases (MMPs), a disintegrin and metalloproteinase with thrombospondin-like motifs (ADAMTS), cathepsins (CTS), and plasminogen activator (PA)/plasmin system enzymes, have been reported to play critical roles in folliculogenesis, tissue remodeling, and ovulation [1,3,29,30,31,32,33]. These critical ovarian processes require the reorganization of the collagen-rich ECM surrounding the follicles, as well as the basement membrane separating granulosa and theca cells [34,35,36,37,38]. In addition, proteases regulate other fundamental processes including apoptosis and autophagy [39,40,41,42,43,44]. Ovarian folliculogenesis is tightly regulated by endocrine, paracrine, and autocrine factors, which affect the growth and maturation of oocytes and surrounding granulosa cells [25,45]. MMPs, including MMP-2, MMP-9, MMP-13, and membrane-type MMP-14 and MMP-16, serve as zinc- and calcium-dependent enzymes that collectively degrade proteinaceous ECM components (proteoglycans, laminin, and collagen) [6,46,47]. Their activities are tightly controlled by tissue inhibitors of metalloproteinases (TIMPs) to maintain the critical MMP/TIMP ratio necessary to regulate cell proliferation, differentiation, migration, and survival throughout follicular development [19,35]. The luteinizing hormone surge triggers the transcriptional activation of key proteases, particularly ADAMTS-1 (a disintegrin and metalloproteinase with thrombospondin motifs) and cathepsin L, which play critical roles in follicle rupture [48,49] (Figure 2). ADAMTS-1 facilitates structural remodeling during folliculogenesis by degrading proteoglycans and maintaining basement membrane integrity, whereas its disruption leads to follicle dysgenesis and impaired ovulation [48,49,50,51,52]. Cathepsin B and L, lysosomal cysteine proteases, regulate granulosa cell apoptosis, proliferation, and steroidogenesis while also modulating autophagy. Cathepsin L activity correlates directly with meiotic progression and embryonic development [53,54]. This network of proteases exhibits specific spatio-temporal expression patterns, and their coordinated action enables the cyclical tissue remodeling essential for follicular growth, ovulation, and corpus luteum formation [11]. Despite their ubiquitous function in both germ and somatic cells, specific roles of proteases in ovarian biology and disease are still far from being understood. This review summarizes the current knowledge on this class of proteins and their functions in follicle dynamics, ovulation, and corpus luteum development, as well as their involvement in ovarian disorders.

## 2. Materials and Methods

We searched databases including PubMed, Web of Science, and Scopus for the following keywords: “Protease” OR “Proteinase” OR “Peptidase, Ovary” OR “Ovarian, Folliculogenesis” OR “Follicle development, Ovarian pathology” OR “Ovarian disease, Metalloprotease” OR “MMP, Serine protease, Cysteine protease, Aspartic protease, ADAM” OR “ADAMTS, Cathepsin, Ovulation, Atresia, Polycystic ovary syndrome” OR “PCOS, Premature ovarian failure” OR “POF, Ovarian cancer”. Inclusion Criteria: Studies focusing on proteases in the ovary, including research on proteases involved in folliculogenesis and ovarian pathologies, and in vivo and in vitro studies in both humans and mammalian models. To ensure relevance while capturing important foundational work, we considered only research articles and reviews published in peer-reviewed journals in the last 30 years. Exclusion Criteria: Studies focusing solely on non-ovarian tissues or without direct relevance to ovarian function or pathology, articles not peer-reviewed, publications older than 30 years, case reports (unless they provide unique insights not available elsewhere), articles not available in English, and conference abstracts.

## 3. Roles of Proteases in Ovarian Follicle Development

### 3.1. Metalloproteinases, Plasminogen Activators, and Their Inhibitors

MMPs, tissue inhibitors of metalloproteinases (TIMPs), and PAs play crucial roles in regulating the remodeling of the ovarian ECM (Table 1) [3,4,6]. MMPs are zinc-dependent endopeptidases that are involved in physiological processes, such as embryonic development, tissue morphogenesis, and angiogenesis, and play critical roles in pathological conditions, including inflammation, cancer, and cardiovascular disease [7,8,9]. MMPs are classified into several groups based on their substrate specificity and structural features: collagenases (MMP1, 8, 13, and 18), gelatinases (MMP2 and 9), stromelysins (MMP3, 10, and 11), matrilysins (MMP7 and 26), membrane-type MMPs (MMP14, 15, 16, 17, 24, and 25), metalloelastase (MMP12), epilysin (MMP28), and enamelysin (MMP20) [8,10,11,14,15,16]. MMPs are secreted as zymogens (pro-MMPs) and their function is regulated at multiple levels, including transcription, zymogen activation, and inhibition by TIMPs [6,15,17]. TIMPs are a family of four proteins (TIMP-1, -2, -3, and -4) that bind to the active site of MMPs in a 1:1 stoichiometry, thereby inhibiting their proteolytic activity [7,16,18,19]. In addition, TIMPs have MMP-independent functions and are involved in cell growth and differentiation, angiogenesis, and apoptosis [20,21,22,23]. The balance between MMPs and TIMPs is critical for maintaining ECM homeostasis and regulating follicle development in the ovary [2,24]. The activation of primordial follicles involves the breakdown of their basement membrane, allowing the proliferation of granulosa cells [25,27,28]. The inhibition of MMP activity results in a significant reduction in primary and preantral follicle numbers [1,2,34,35]. MMP2 and MMP9 are expressed throughout folliculogenesis in both granulosa cells and oocytes [12,29,30,31,45]. TIMP-1 and TIMP-2 are also expressed in the granulosa cells and oocytes of primordial and primary follicles in humans [30]. Their expression decreases during the transition from primordial to primary follicles, suggesting that a reduction in TIMP activity may be necessary for follicular activation [30]. TIMP-1-deficient mice show increased MMP activity, particularly MMP2, and MMP9, and display an increased number of primary and preantral follicular compared to wild-type mice [36,37,38,55] (Table 2).

In addition to MMPs and TIMPs, PAs play an important role in the ovary [56]. PAs convert inactive plasminogen into plasmin, which is involved in the degradation of the ECM proteins, including fibrin, fibronectin, and laminin [57,58,59]. The PA system consists of a tissue-type plasminogen activator (tPA) and a urokinase-type plasminogen activator (uPA). PAs are regulated by protease inhibitors α2-antiplasmin and α2-macroglobulin, and plasminogen activator inhibitors (PAIs), which belong to the serine proteinase inhibitor (serpin) gene superfamily and include PAI-1, PAI-2, PAI-3, and the protease nexin I [39,40,41,42,43,57,60]. In the ovary, PAs are produced in granulosa cells and oocytes, whereas the majority of PAI-1 is produced within the theca (interstitium) [4,61,62,63]. uPA and tPA are upregulated during follicle activation and maturation, and their expression is regulated by gonadotropins, growth factors, and cytokines (Table 1) [57,59,64,65,66]. Li et al. showed that TGFα treatment increases uPA levels and activity in hen granulosa cells across all the follicle stages [66]. Similarly, FSH induces both uPA and tPA, whereas IL-1β suppresses their activity in vitro [67]. In rat ovaries, Hurwitz et al. also reported that IL-1β functions as an inhibitor of the PA system [68].

Other serine proteases that have been reported in the regulation of folliculogenesis include LONP1, FURIN, PAPPA, and matriptase (Table 2). The mitochondrial LONP1 is a multifunctional protease involved in mitochondrial quality control including oxidized protein degradation, protein folding, and mitochondrial DNA copy number homeostasis. The oocyte-specific ablation of *Lonp* using *Gdf9-cre* or *Zp3-cre* results in female infertility due to impaired follicle development, loss of the ovarian reserve, and progressive oocyte death ([69]. *FURIN* encodes a transmembrane serine protease localized in the Golgi apparatus, endosomes, and plasma membrane. The conditional ablation of *Furin* using *Gdf9-cre* or *Zp3-cre* leads to female infertility due to arrested folliculogenesis at the secondary follicle stage [70]. *PAPPA* encodes an extracellular metalloprotease, and *Pappa* knockout females exhibit decreased litter size and ovulatory capacity, which was attributed to the decreased bioavailability of insulin-like growth factor [71,72,73]. Matriptase, encoded by *Tmprss6*, is a type II transmembrane serine protease that regulates iron homeostasis by cleaving cell surface proteins associated with iron absorption. *Tmprss6*-null females show a severe delay in follicle maturation, likely due to a significant decrease in plasma iron levels [74]. Notably, defective follicle development and female infertility can be reproduced by a low-iron diet [75].

**Table 1:** Protease functions during ovarian physiology and follicular development.

Protease	Mechanism of Regulation	Specific Stage	Function	Species	Reference
MMP1	Increased expression following hCG administration	Preovulatory	Degradation of collagenous ECM	Rhesus monkey	[18,76]
MMP2 and MMP9	Increased in the granulosa and thecal cells of atretic follicles during proestrus and in corpus luteum during metestrus	Preovulatory follicles	ECM remodeling	Guinea pigs	[45]
MMP2, and MMP9	Localized to the oogonium/oocyte cytoplasm and surface epithelium	Folliculogenesis	ECM remodeling during gonadal development and cell–matrix interactions	Human	[77]
MMP1 and MMP13	Expression increased in response to LH surge	Preovulatory	Degradation of collagenous ECM	Bovine	[78]
MMP1, MMP2, MMP3, MMP9, MMP13	Increased in mRNA expression by gonadotropins	Prehierarchical white (WFs), yellowish (YFs), and preovulatory follicles	Involved in the atresia of the early stage of follicle while not participating in the regulation of advanced stage atresia	Chicken	[24,79]
MMP1, MMP3, and MMP9	Increased MMP1 and MMP3 expression levels in granulosa	Folliculogenesis	MMP9 induced by TGFB1; MMP1and MMP3 stimulated by FSH, LH, P4, and E2	Chicken	[80]
MMP10 and MMP11	Expression patterns changes following hCG administration	Ovulation and luteogenesis	Mmp10 mRNA was increased and MMP11 decreased in granulosa and theca cells during Ovulation	Human and Rats	[81]
MMP13, MMP14, MMP16, ADAMT1	Increased expression in cumulus cells following hCG administration	Ovulation and luteogenesis	Migratory phenotype of the cumulus–oocyte complex at the time of ovulation	Rat	[82]
MMP19	Localized to granulosa and theca-interstitial cells with temporal increases following hCG administration	Preovulatory follicles	ECM remodeling and tissue degradation	Mouse, Rat, Bovineand Human	[76,82,83]
MMP2, MMP9, TIMP-1, and TIMP-2	The ratio of MMP-2/TIMP-2 decreased in small antral follicles; the MMP-9/TIMP-1 ratio increased in large-preovulatory follicles	Preovulatory follicles	Tissue reorganization during ovulation	Equine	[29]
TIMP-2 and TIMP-3	Increased transcript abundance of TIMP-2 in yellow atretic follicles; decreased mRNA expression of TIMP-3	Prehierarchical white (WFs), yellowish (YFs), and preovulatory follicles	Involved in the atresia	Chicken	[24,79]
TIMP4	Increased significantly during the luteinization process of granulosa cells	Localized to the theca of antral and preovulatory follicles and adjacent ovarian stroma	Maintenance of luteal function	Mice, Rat	[84,85]
tPA and uPA	Activity increased during the periovulatory period	Granulosa and theca cells	Conversion of plasminogen to plasmin during ovulation	Rat	[66,86,87]
tPA and uPA	TNFα suppressed FSH-stimulated tPA activity but potentiated FSH-induced uPA activity in undifferentiated granulosa cells	Undifferentiated granulosa cells of preantral and antral follicles	Follicular wall remodeling during ovarian follicular development	Rat	[67,88]
PAI-1 and PAI-2	mRNAs upregulated after the gonadotrophin surge	PAI-1 localized to the thecal layer of preovulatory follicles. PAI-2 localized to the granulosa cell	Control plasminogen activator activity associated with ovulation and early corpus luteum formation.	Bovine	[89]
CTSL	Expression increased following hCG administration	Oocyte meiosis, Preovulatory to ovulation	Degradation of the follicular wall	Rat, Rhesus monkey, Bovine	[54,76]
CTSB	Expression increased following hCG administration, Autophagy induction	Preovulatory to ovulation	Regulation of follicular development	Mice, Bovine	[53,90]
CTSB, K, L, and H	Expressed in germinal epithelium throughout the estrous cycle	Oocytes and granulosa cells of primordial, primary follicles and corpus luteum	Degradation of extracellular matrix	Mice	[91,92]
Kallikreins	Response to steroid hormones (androgens and estrogens); various expression patterns with eCG/hCG stimulation	Primordial to ovulation	Proteolytic processing of growth factors and hormones; angiogenesis	Rat	[93,94]

### 3.2. Cathepsins

Cathepsins are classified into three main groups based on their catalytic site residues: cysteine cathepsins (Types B, C, F, H, K, L, O, S, V, W, and X), aspartic cathepsins (Types D and E), and serine cathepsins (Type G) [95,96]. In the ovary, cathepsins are expressed in oocytes, granulosa, and theca cells, and their expression is regulated by gonadotropins and growth factors (Table 1) [92].

- Cathepsin B (CTSB): CTSB can function both as endo- and exo-(carboxy) peptidase [97,98]. CTSB has been identified as a critical regulator of ovarian reserve maintenance in mice [99]. The inhibition of *Ctsb* by myricetin significantly increased the number of primordial and primary follicles, suggesting a role in follicle activation. This effect seems mediated by the inhibition of autophagy and upregulation of the IGF1R and AKT-mTOR pathways [99]. Similarly, Liang et al. reported that the inhibition of CTSB activity preserved oocyte quality and enhanced developmental competence by mitigating age-related mitochondrial dysfunction and oxidative stress [100]. Chen et al. reported that the silencing of *Ctsb* in mouse granulosa cells decreased apoptosis by downregulating *TNF-α*, *Casp8*, and *Casp3* while upregulating *Bcl2* expression [53]. *Ctsb* knockdown also increased granulosa cell proliferation by activating the *p*-Akt and *p*-ERK pathways [53]. Komatsu et al. reported that Stefin A, an inhibitor of CTSB, blocked the activation of primordial follicles in mouse newborn ovaries *in vitro* [101]. In the follicle fluid of pregnant women undergoing ICSI, Bastu et al. found higher levels of CTSB compared to non-pregnant patients [102].
- Cathepsin L (CTSL): *Ctsl* is involved in the activation of primordial follicles adjacent to ovulatory follicles, and its inhibition results in a significant reduction in growing follicle numbers [92]. *Ctsl* expression was detected in large cuboidal cells of small, developing corpora lutea, suggesting possible roles in corpus luteum function [91,103]. Ezz et al. showed that CTSL regulates oocyte meiosis, and its supplementation improves oocyte quality and early embryo development in the bovine [54] (Table 2).
- Cathepsin S (CTSS): Song et al. reported that *Ctss* overexpression significantly increased progesterone (P4) and estrogen (E2) production by upregulating *Star* and *Cyp19a1* in rabbit granulosa cells [104,105]. The overexpression of *Ctss* also increased granulosa cell proliferation while decreasing apoptosis by enhancing the expression of *Pcna* and *Bcl2*. Conversely, *Ctss* knockdown significantly decreased the secretion of P4 and E2 while increasing apoptosis [104].

**Table 2:** Effects of protease gene knockout or protease inhibition on follicle development in model organisms.

Protease	KO/Inhibitor	Effect on Follicular Development	Specific Stage	Molecular Mechanism	Localization	Species	Reference
MMP1, MMP9, MMP10, and MMP19	Inhibitor (GM6001)	Reduced ovulation rate	Preovulatory to ovulation	Degradation of the follicular wall	Granulosa and theca cells	Rhesus monkey	[76]
MMP2	Inhibitor (ZK158252)	Inhibited hCG-induced ovulation and MMP-2 activation	Preovulatory to ovulation	Leukotriene B4-receptor antagonism	Ovarian follicles	Rat	[106]
MMP1, MMP2, and MMP3	Inhibitor (GM6001)	Reduction in CL and E2 with GM6001	Preovulatory to ovulation	Inhibits MMP activity in photostimulated ovaries	-	Hamster	[107,108]
MMP10	Inhibitor AG1478	Up-regulation of Mmp10 by LH.	Ovulation and luteinization.	Suppressed the induction of Mmp10 mRNA	Granulosa cells	Rat	[81]
TIMP-1	KO	Increased number of primary and preantral follicles	Primordial to primary/preantral	Regulation of MMP activity	-	Rodent	[38,109]
CTSB	Inhibitor (Myricetin)	Increased oocyte reserve	Primordial to primary	Inhibition of autophagy and upregulation of the IGF1R and AKT-mTOR pathways	Oocytes	Mouse	[99]
CTSL	siRNA	Enhanced fertilization capability and blastocyst formation	Oocytes	Increasing mitochondrial function, reducing accumulated ROS, lowering apoptosis, and recovering lysosome capacity	Oocytes	Mouse	[91]
CTSL	KO	Reduced ovulation rate	Preovulatory to ovulation	Degradation of the follicular wall	Granulosa and theca cells	Mice	[48]
CTSL	rCTSL supplementation	Regulated oocyte meiosis during maturation and early embryo development	Oocyte maturation	Meiotic regulation	Oocytes	Bovine	[54,76]
CTSB	Stefin A	Blocked activation of primordial follicles	Primordial follicles	17β-estradiol increased Stefin A mRNA expression and inhibited follicle development	-	Mouse	[101]
ADAMTS1	KO	Lower numbers of mature follicles and impaired ovulation	Antral to ovulation	Maintenance of follicular basement membrane integrity	Granulosa cells	Mouse	[110,111]
ADAMTS1	KO	Failure of ovulation and fertilization	Preovulatory to ovulation	Expansion of cumulus–oocyte complexes (COCs)	COCs	Mouse	[49,52,112]
ADAMTS9	KO	Ovarian malformation and inability to ovulate	Primordial to ovulation	-	-	Zebrafish	[113]
LONP1	Oocyte-specific KO	Impaired follicular development and progressive oocyte death	Primordial to antral	Regulation of mitochondrial function	Oocytes	Mouse	[69]
FURIN	Oocyte-specific KO	Arrested oogenesis at early secondary follicles	Primary to secondary	-	Oocytes	Mouse	[70]
PAPPA	KO	Decreased litter size and ovulatory capacity	Antral to ovulation	Regulation of IGF bioavailability	-	Mouse	[71,72,73]
TMPRSS6	KO	Retardation in ovarian maturation	Primordial to antral	Regulation of iron homeostasis	-	Mouse	[74]
tPA and uPA	Inhibitor (PAI-1)	Significantly reduced ovulation rate	Preovulatory to ovulation	ECM degradation	-	Rat, Human	[87,114,115,116,117]
PA	Protease nexin-1 (SerpinE2)	tPA activity higher in cells from small follicles; SerpinE2 levels higher in large follicles	Antral and basal granulosa cells	SerpinE2 secretion regulated at the transcriptional level	Granulosa cells	Bovine	[41]

## 4. Role of Proteases in Antral Follicle Development and Ovulation

### 4.1. Metalloproteinases, Plasminogen Activators, and Their Inhibitors

Ovulation is a complex process involving the rupture of the preovulatory follicle and the release of the oocyte [118,119]. A critical step in this process is the degradation of the basal membrane and ECM surrounding the mature follicle, which are rich in collagen, laminin, and fibronectin [2,57]. MMP2 and MMP9 are expressed in the granulosa and theca cells of preovulatory follicles of both rodents and humans, and their levels increase as the follicles approach ovulation [1,31,34,46,81,118]. Conversely, the inhibition of their activity significantly reduces ovulation rates [31,120,121,122].

Several studies have analyzed changes in Mmp expression following the administration of human chorionic gonadotropin (hCG), which mimics the luteinizing hormone (LH) surge necessary for ovulation. In rhesus monkeys, MMP1 was found to increase following hCG treatment [18]. *Mmp11* decreased in both humans and rats [81], whereas *Mmp13* increased in bovines [78], but not in humans [83], and *Mmp19* increased in mice [63], rats [123], and humans [83]. ADAMTS1 is a secreted metalloproteinase expressed in the granulosa cell layer of mature follicles in the ovary [51,111,112]. LH stimulates the expression of *Adamts1* in the granulosa cells of the preovulatory follicles and is sustained in a progesterone-dependent manner [48]. *Adamts1*-null female mice display lower numbers of growing follicles and impaired ovulation due to mature oocytes remaining trapped within the antral follicles [110,111]. In *Adamts1* knockout mice, the development of the ovarian medullary vascular network and lymphatic system were severely delayed, and the expansion of cumulus–oocyte complex (COCs) was reduced, causing lower ovulation and fertilization rates [49,52,112]. These findings highlight a role for *Adamts1* in maintaining the structural integrity of follicle basement membranes and supporting lymphangiogenesis, therefore providing new mechanistic insight into ovarian development and disease.

PAs and their inhibitors PAIs are key regulators of the proteolytic cascade involved in ovulation [57,64,124]. During follicle growth, granulosa and theca cells produce tPA and uPA, which facilitate the breakdown of the surrounding basement membrane and the subsequent release of the oocyte [57,59,125,126,127]. Consistent with these observations, PAI-1 expression was reported to decrease during follicle maturation in the human ovary, allowing higher PA activity [128]. Similarly, in rats and porcine ovaries, tPA and uPA activity increased during the periovulatory period, peaking just before ovulation [86,87,114,124,129]. In addition to tPA and uPA, kallikreins are serine proteases that participate in plasminogen activation and promote vascularization [130,131,132,133]. Accumulating evidence suggests that kallikrein expression is regulated by steroid hormones (Table 1). Estrogens stimulate the secretion of KLK1, KLK10, KLK11, and KLK14, whereas androgens trigger the secretion of KLK3 [134,135]. Interestingly, combined stimulation with both androgens and estrogens downregulates KLK3 expression, consistently with the antagonistic effect of estrogens on androgen receptor activity [135,136,137,138]. KLK5 and KLK6 were found to be involved in the proteolytic processing of growth factors and hormones essential for follicular development and ovulation [94,139,140,141].

### 4.2. Cathepsins

Several studies have reported the involvement of both CTSB and CTSL in regulating ovulation [100,142,143]. In bovine ovaries, Balboula et al. found an increased expression of CTSB in follicle granulosa and theca cells following hCG administration, whereas inhibition of CTSB activity resulted in a significant reduction in ovulation rates [144,145]. The expression of *Ctsl* increases in the granulosa cells of mouse preovulatory follicles following PMSG and hCG administration [48]. In addition, Sriraman et al. found an increased expression of CTSL in the granulosa cells of human preovulatory follicles, with levels peaking just before ovulation [142]. García et al. proposed that the transient expression of progesterone receptor (PR) in human granulosa cells of the preovulatory follicle may play a role in the activation of CTSL [146]. Interestingly, Zhang et al. demonstrated a significant increase in *Ctsl* levels in oocytes of aged mice (8–9 months and 11–12 months), and found that the overexpression of *Ctsl* in the oocytes of young mice (6–8 weeks) substantially diminished their quality, which was restored upon *Ctsl* inhibition [91].

## 5. Role of Proteases in Corpus Luteum Formation and Function

Following ovulation, the remainder of the ruptured follicle transforms into the corpus luteum (CL). The CL is a transient endocrine gland that is necessary to sustain pregnancy through the production of progesterone, which prepares the uterus for implantation and pregnancy [147,148,149]. If fertilization does not occur, the CL degenerates leading to the initiation of a new cycle. Dramatic ECM remodeling and angiogenesis are involved in the development, activity, and regression of the CL. MMP2, MMP13, and MMP14 seem to be involved in the regulation of CL dynamics [46,92]. The degradation of the ECM by plasmin allows for the rapid remodeling and vascularization of the CL, which is essential for establishing its function [2,115,135,150]. Wahlberg et al. investigated the formation and function of the CL in plasminogen (*Plg*)-deficient mice, with or without the administration of galardin, a broad-spectrum synthetic MMP inhibitor [151]. The study revealed that CL formation occurred in *Plg*-deficient mice, galardin-treated wild-type mice, and galardin-treated *Plg*-deficient mice, demonstrating that neither the plasminogen activator nor the MMP system is necessary for CL formation. However, serum progesterone levels in *Plg*-deficient mice were reduced by approximately 50%, and galardin treatment did not further decrease progesterone concentrations. These findings suggest that plasmin, but not MMPs, seems to play a significant role in maintaining luteal function, possibly through the proteolytic activation of growth factors and other paracrine factors [151].

## 6. Role of Proteases in Ovarian Disease

**Figure 3:**
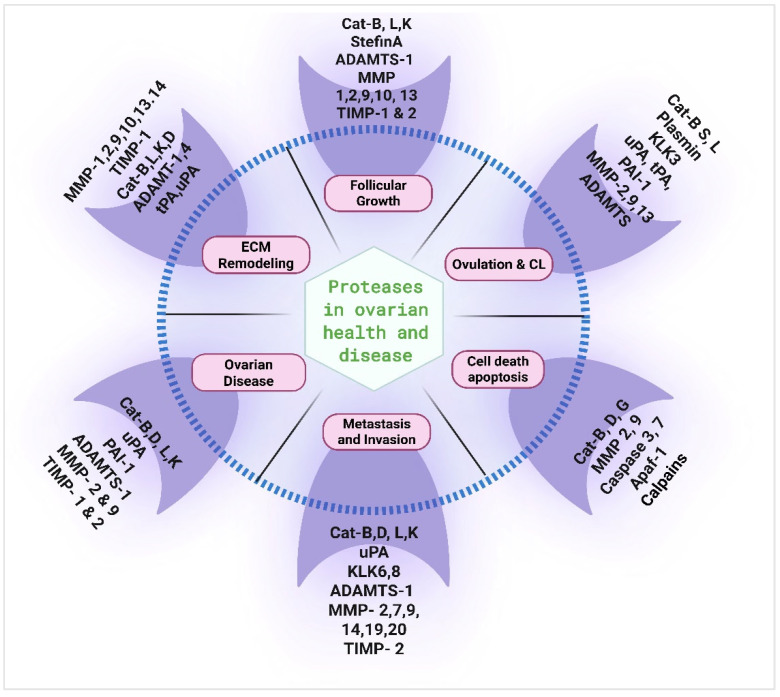
Roles of proteases in ovarian health and disease. Matrix metalloproteinases (MMPs), tissue inhibitors of metalloproteinases (TIMPs), plasminogen activators (PAs), plasminogen activator inhibitors (PAIs), ADAMTS, cathepsins, and kallikreins are involved in critical physiological processes of the ovary, including folliculogenesis, extracellular matrix (ECM) remodeling, apoptosis, ovulation, corpus luteum (CL) development. The dysregulation of proteases can lead to several pathological conditions including cancer, polycystic ovary syndrome (PCOS), primary ovarian insufficiency (POI), and ovulatory irregularities.

Due to the critical role proteases play in numerous biological processes, including cell proliferation, migration, and programmed cell death, the dysregulation of their function can lead to several ovarian pathologies (Figure 3).

### 6.1. Ovarian Cancer

Ovarian cancer is the fifth leading cause of cancer death in females with long-term survival rates of 20% or less in advanced stages III-IV [152,153,154]. The dysregulation of protease activity has been linked to ovarian cancer pathogenesis (Table 3) [33,155,156].

- MMPs and TIMPs: MMPs participate in several processes that are involved in ovarian cancer progression, including the degradation of the ECM, the promotion of angiogenesis, and the induction of epithelial–mesenchymal transition (EMT) [6,35,46,157,158,159]. Several studies have shown the upregulation of MMPs, such as MMP2 and MMP9, in ovarian cancer tissues compared to normal or benign ovarian tissues, and their expression levels correlate with clinical stage, tumor invasiveness, and metastatic potential [155,157,160,161]. Tumor-derived MMP2 and MMP9 expression has been identified as a negative prognostic indicator in ovarian cancer patients, predicting lower overall survival rates [162,163,164,165,166]. Ovarian cancer cells (Ovcar3) treated with an activator of the PKC pathway, phorbol-12-myristate 13-acetate (PMA), increased *MMP7* and *MMP10* mRNA [167,168]. MMP14 was shown to activate pro-MMP2 to MMP2, playing a role in the development of vasculogenic-like networks and matrix remodeling by aggressive ovarian cancer cells [168,169,170]. MMP1 activates PAR1, inducing the secretion of angiogenic factors in ovarian carcinoma cells [171]. MMP3 is involved in the estradiol-induced migration and invasion of SKOV3 ovarian cancer cells via the PI3K/Akt/FOXO3 pathway [172]. MMP7 promotes the invasion and metastasis of ovarian cancer cells by activating gelatinases and through the MAPK/ERK and JNK pathways [173,174]. MMP8 upregulates IL-1β, whose expression levels correlate with tumor grade and poor prognosis [175]. The *MMP12* 82A/G polymorphism has been associated with increased susceptibility to ovarian cancer [176,177], and MMP13 in ascitic fluids of ovarian cancer patients has been identified as a potential marker for disease risk and survival outcomes [178]. Taken together, these studies underline the association between the dysregulation of MMP expression and activity and ovarian cancer.
  In addition to MMPs, several TIMPs, including TIMP1 and TIMP3, have been found upregulated in ovarian cancer [179,180]. However, Davidson et al. found decreased TIMP levels alongside increased MMP2 in ovarian cancer [181]. These seemingly contradictory results highlight the complex mechanisms involved in ovarian cancer and show how dysregulation of the MMP/TIMP balance may have a more significant impact than the overexpression of a single class of proteins [157,182]. In addition, there are several possible explanations for the conflicting findings of increased levels of both MMPs and their inhibitors: (1) TIMPs regulate processes independent of their protease inhibitory activity, including cell growth, migration, and angiogenesis [183]; (2) the stoichiometric balance between MMPs and TIMPs may be more critical than absolute levels, and elevated TIMP levels may sometimes be insufficient to counteract excessive MMP activity in aggressive cancers [184]; (3) TIMPs have been found to activate MMPs in certain instances [19]; and (4) different tissue compartments may have varying MMP ratios, allowing MMPs to remain active in specific microenvironments despite elevated TIMP levels [185].
- The PA and PAI system: In vitro analyses have shown that uPA is highly expressed in several types of cancer cells, including ovarian cancer [186,187,188,189,190,191,192,193]. The overexpression of uPA and PAI-1 was found in more than 75% of primary ovarian carcinomas, and in most metastatic epithelial ovarian cancer (EOC) [194]. Further, Kenny et al. reported that in vitro and in vivo treatments with a uPA receptor (uPAR) antibody inhibited ovarian cancer cell invasion, migration, and adhesion by inhibiting α5-integrin and decreasing the expression of urokinase, uPAR, β3-integrin, and fibroblast growth factor receptor-1 [195]. High levels of PAI-1 have been associated with poor clinical outcomes in ovarian serous carcinoma [187,196]. In ovarian cancer cells, PAI-1 inhibition resulted in cell cycle arrest and decreased proliferation, and, in xenograft models, significantly reduced peritoneal dissemination [196]. Similarly, PAI-1 silencing in SKOV3 cells disrupted the platelet-induced upregulation of the genes involved in proliferation and ECM remodeling [197]. At the molecular level, some reports suggest that PAI-1 inhibits cell adhesion and migration by blocking vitronectin (VN) binding to integrins or by displacing uPAR from VN in the extracellular matrix [198,199]. However, other studies have shown that PAI-1 can enhance cancer cell adhesion [200,201].
  As for MMPs/TIMPs, it is unclear why the upregulation of both uPA and PAIs correlates with cancer progression and poor clinical outcomes. Several mechanisms may explain this apparent contradiction: (1) PAI have additional functions beyond uPA inhibition, including activation of pathways that promote tumor growth, angiogenesis, and cell detachment [202,203,204]; (2) the PAI-1/uPA/uPAR complex can be internalized and recycled, potentially leading to increased uPAR on the cell surface and enhanced invasiveness [205]; (3) PAI-1 can elicit inflammatory responses and immune cell recruitment in the tumor microenvironment, potentially promoting a pro-tumorigenic milieu [206]. Overall, these findings suggest that PAI function is context-dependent and highlight the complex regulation of the PA/PAI system in ovarian cancer.
- Cathepsins: Cathepsins and their inhibitors cystatins have also been associated with ovarian cancer. Liu et al. showed that CTSB and its binding proteins AMBP and TSRC1 modulated TNF-induced apoptosis in ovarian cancer cells [207]. Additionally, *Ctsl* knockdown inhibited proliferation, invasion, and tumor growth both in vitro and in vivo, while *Ctsl* overexpression had the opposite effects [208,209]. In malignant serous tumors, cystic fluid levels of CTSB, CTSL, and their inhibitor Cystatin C (Cst3) were significantly elevated compared to benign serous tumors [210]. Gashenko et al. found significantly increased levels of procathepsin B, cystatin B (CstB), and Cst3 in serum and ascite fluids of ovarian cancer patients compared to controls, suggesting their possible use as disease biomarkers [211]. Nishikawa et al. found significantly elevated levels of Cst3, but not CTSB, in ovarian cancer compared to benign samples and healthy controls [212]. Interestingly, invasion assays showed that the inhibition of Cst3 or CTSB suppressed cancer cell invasion in a dose-dependent manner [212]. Once again, some of these findings appear contradictory. Elevated CysC in cancer may represent a compensatory mechanism to control excessive cathepsin activity [213]. In addition, similar to other protease inhibitors, Cst3 may have additional functions including the regulation of immune response and cell signaling [214]. Furthermore, changes in the balance between cathepsins and cystatins may be more important than absolute expression levels of either protein [215]. Finally. Cst3 primarily regulates extracellular cathepsin activity, while pro-tumorigenic effects of CTSB may be, at least in part, intracellular [216].

Xu et al. reported elevated levels of serum CTSK in ovarian cancer patients compared to healthy controls [217]. CTSD was also found associated with ovarian cancer, with epithelial CTSD expression more common (65.1%) in ovarian tumors with low malignant potential (LMP) compared to invasive tumors (43.7%) [218,219]. CTSD was also found to significantly increase the proliferation and migration of human omental microvascular endothelial cells, and the knockdown of *CTSD* was exhibited to be able to suppress migration and invasion in an EOC cell line [220,221]. In addition, the inhibition of CTSS induced apoptosis in cancer cells by downregulating Bcl-2 and c-FLIP [222].

Another serine protease, Hepsin is overexpressed in several cancers including ovarian cancer, but its specific role remains to be elucidated [223,224,225,226]. Miao et al. reported that membrane-associated Hepsin localized at desmosomal junctions with its putative proteolytic substrate hepatocyte growth factor (HGF), and showed that Hepsin overexpression promoted ovarian tumor growth in a mouse model [227]. The dysregulation of Hepsin expression could disrupt the integrity and function of epithelial barriers. In addition, the Hepsin-mediated cleavage of substrates including pro-HGF and pro-uPA could promote metastasis [228,229]. Despite a role in mediating cancer formation and progression, sosme reports suggest that high levels of Hepsin could have antitumor effects by reducing oncogenic signaling and increasing autophagy [230]. It is possible that specific functions in cancer depend on the microenvironment or disease stage.

**Table 3:** Proteases in ovarian cancer.

Protease	Finding in Ovarian Cancer	Localization	Species	Prognostic Value	Role	Reference
MMP1	Activates PAR1	Ovarian carcinoma cells	Human, Epithelial ovary cell lines	Not reported	Induces the secretion of angiogenic factors	[231,232,233,234]
MMP2	Upregulated in ovarian cancer tissues compared to normal/benign ovarian tissues	Ovarian cancer tissue, epithilial, stroma	Human	Negative prognostic indicator with lower overall survival rates	Degrades ECM, promotes angiogenesis, and induces EMT	[161,162,163,182,235,236,237]
MMP3	Involved in estradiol-induced migration and invasion	SKOV3 ovarian cancer cells	Human cell line	Not reported	Mediates estrogen-induced cancer progression via PI3K/Akt/FOXO3 pathway	[172]
MMP7	Promotes invasion and metastasis	Ovarian cancer cells	Human cell line	Not reported	Acts through MAPK/ERK and JNK pathways; activates gelatin enzymes	[174]
MMP8	Upregulates IL-1β	Ovarian cancer tissue	Human	Correlates with tumor grade and poor prognosis	Promotes inflammatory microenvironment	[175]
MMP9	Upregulated in ovarian cancer tissues	Ovarian cancer tissue	Human	Correlates with clinical stage, tumor invasiveness, and metastatic potential	Degrades ECM, promotes angiogenesis, and induces EMT; cleaves fibronectin and type IV collagen	[23,165,165,238,239]
MMP10	Increased expression with PKC pathway activation	Ovarian cancer cells (Ovcar3, EOC)	Human cell line	Not reported	Regulated by PKC pathway, Wnt signaling	[167,240]
MMP11	Overexpression in stromal cells	Ovarian carcinomas	Human	Overexpression not correlates with survival.	Tumor progression	[241]
MMP12	82 A/G polymorphism associated with increased susceptibility	Genetic study	Human	Not reported	Genetic predisposition factor	[176,177]
MMP13	Elevated in ascitic fluids	Ascitic fluid	Human	Potential marker for disease risk and survival outcomes	Not reported	[178,179]
MMP14	Activates pro-MMP2 to MMP2	Ovarian cancer cells	Human	Associated with vasculogenic-like networks	Matrix remodeling; activates pro-MMP2	[82,168,242]
TIMP1	Upregulated in ovarian cancer	Ovarian cancer tissue	Human	Not reported	Complex: May have MMP-independent roles in cell growth, migration, and angiogenesis	[180,181]
TIMP3	Upregulated in ovarian cancer	Ovarian cancer tissue	Human	Not reported	Complex regulatory roles beyond MMP inhibition	[243,244,245]
uPA	Highly expressed in cancer cells; overexpressed in >75% of primary ovarian carcinomas and metastatic EOC samples	Ovarian cancer cells	Human and cell lines	Associated with invasion and metastasis	Promotes invasion, migration, and adhesion	[187,188,189,190,191]
PAI-1	High levels in ovarian serous carcinoma	Ovarian cancer tissue	Human, cell lines, and xenograft models	Associated with poor clinical outcomes	Complex: Inhibits cell adhesion by blocking vitronectin; disrupts platelet-induced gene upregulation	[189,197,198]
CTSB	Modulates TNF-induced apoptosis; elevated in cystic fluid and serum	Cystic fluid, serum, and cancer cells	Human	Serum procathepsin B significantly elevated compared to healthy controls	Binding proteins AMBP and TSRC1 involved in TNF-induced apoptosis	[210,211,212]
CTSL	Overexpressed; knockdown inhibits proliferation, invasion, and tumor growth	Cancer cells	Human, cell lines, and mouse models	Associated with paclitaxel resistance	Promotes proliferation and migration; confers chemoresistance	[208,209]
CTSS	Inhibition stimulates TRAIL-induced apoptosis	Cancer cells	Human	Not reported	Downregulation of Bcl-2 and Cbl-mediated c-FLIP by ROS-mediated p53 expression	[222]
Cst3	Elevated in malignant tissues, serum, and cystic fluid	Malignant tissue, Serum, Cystic fluid	Human	Elevated in ovarian cancer compared to benign samples	Complex: May represent failed compensatory mechanism; has additional immune and signaling functions	[210,212,213,214,215]
CTSK	Overexpressed in peritoneal metastatic ovarian carcinomas; elevated serum levels	Peritoneal metastases, Serum	Human	Potential biomarker	Associated with peritoneal metastasis	[217,246]
CTSD	Expression more common (65.1%) in tumors with low malignant potential vs. invasive tumors (43.7%); promotes the proliferation and migration of endothelial cells	Epithelial cells, Stromal cells	Human and cell lines	Independent prognostic factor for disease-free survival in invasive ovarian cancer	Pro-angiogenic and pro-metastatic role via ERK1/2 and AKT activation; correlates with microvessel density	[218,219,220,221]
Hepsin	Overexpressed in ovarian cancer	Desmosomal junctions	Human and mouse model	Not reported	Cleaves HGF and pro-uPA; localizes with substrate HGF; disrupts epithelial barriers	[223,224,225,227,229]

### 6.2. Polycystic Ovary Syndrome (PCOS)

PCOS is an endocrine disorder characterized by ovulatory dysfunction, excess of androgen, and polycystic ovaries, often leading to infertility, insulin resistance, and cardiovascular disease [247,248,249,250,251].

- MMPs and TIMPs: MMPs and TIMPs have been associated with the pathogenesis of PCOS (Table 4) [252]. It has been reported that MMP2 and MMP9 concentrations are elevated in the follicular fluid of patients with PCOS compared to healthy controls [252,253]. The increased MMP activity was associated with higher levels of androgens, insulin resistance, disrupted follicular development, and ovulatory dysfunction [6,253,254]. Consistent with these observations, it was found that the granulosa cells of women with PCOS express fewer MMP inhibitors TIMP-1 and TIMP-2 compared to healthy controls [252,255]. Recently, Butler et al. reported that women with PCOS showed significantly elevated MMP9 [254]. Interestingly, the ratios of MMP9 to all TIMPs were significantly higher in the PCOS group, while MMP17/TIMP-1 and MMP17/TIMP-2 were lower. Higher expression of Mmp2/9 was also observed in antral follicles compared to the preantral follicle and primordial follicle of a Letrozole-induced PCOS rat model [256].
- The PA and PAI system: Elevated PAI-1 levels in plasma have been reported in patients with PCOS compared to controls (Table 4) [257,258,259,260,261,262]. However, findings regarding PAI-1 distribution within ovarian tissue have been inconsistent. Devin et al. reported increased PAI-1 in granulosa cells of cystic and atretic follicles in mouse models of PCOS [263]. Atiomo et al. detected PAI-1 in granulosa and theca cells without significant differences between PCOS and control ovaries, whereas other authors reported increased PAI-1 expression in follicular fluid from patients with PCOS [257,264,265]. Genetic predisposition seems to contribute to PAI-1 dysregulation in PCOS, which has been reported associated with the 4G/4G and 4G/5G genotypic subtypes in the PAI-1 promoter region, leading to increased protein levels [266].
  Kelly et al. observed increased tPA antigen levels inversely correlating with insulin resistance, whereas Tarkun et al. found a direct correlation of PAI-1 levels, even in lean PCOS women [197,259]. Orio et al. reported elevated PAI-1 activity independent of obesity, while Sahay et al. found a correlation with both insulin resistance and obesity [260,267]. Ma et al. provided mechanistic insight through a mouse model demonstrating that PAI-1 deficiency prevented diet-induced obesity and insulin resistance [268,269]. Finally, Ibrahim et al. reported the presence of KLK2 in the serum of women with PCOS in association with hirsutism, but the nature of this relationship remains unclear [270].
- Cathepsins: The downregulation of CTSD has been reported in the ovaries of patients with PCOS [271]. CTSD downregulation may contribute to the abnormal follicle development associated with PCOS, leading to ovulatory dysfunction and infertility. Dawood et al. found significantly increased levels of CTSS, among patients with PCOS compared to healthy females [272]. Additionally, genetics may also play a role as CTSB polymorphisms have recently been associated with PCOS risks [273].

**Table 4:** Protease dysregulation in PCOS.

Protease	Finding in PCOS	Localization	Species	Proposed Pathogenic Role	Reference
MMP2 And MMP9	Elevated in follicular fluid of patients with PCOS compared to healthy controls	Follicular fluid and serum	Human	Associated with higher levels of androgens, insulin resistance, disrupted follicular development, and ovulatory dysfunction. Associated with higher MMP9/TIMP ratios, ECM remodeling, and follicular development	[252,253,256]
MMP2/9	Higher expression in antral follicles compared to preantral and primordial follicles	Ovarian follicles	Rat (Letrozole-induced PCOS model)	ECM remodeling in PCOS ovaries	[256]
MMP17	Lower MMP17/TIMP-1 and MMP17/TIMP-2 ratios in PCOS	Serum	Human	ECM remodeling	[254]
TIMP-1 and TIMP-2	Decreased expression in granulosa cells of women with PCOS	Granulosa cells	Human	Excessive ECM degradation	[255,274]
PAI-1	Elevated in plasma, granulosa cells, and follicular fluid; homogeneous distribution throughout PCOS ovaries	Plasma, granulosa cells, follicular fluid, and theca cells	Human and mouse (PCOS model)	Associated with insulin resistance; higher expression in 4G/4G and 4G/5G genotypes	[260,262,263,264,266,267,268,269]
Plasminogen	Uniquely present in small follicles of PCOS ovaries	Small follicles	Human	Altered proteolytic activity in early follicular development	[258,259,261]
tPA	Increased antigen levels inversely correlating with insulin resistance	Plasma	Human	Associated with insulin resistance	[275]
KLK2/3	Present in the serum of women with PCOS in association with hirsutism	Serum	Human	Associated with androgsen excess and hirsutism	[270]
CTSB	CTSB polymorphisms contribute to PCOS pathogenesis	Blood	Human	rs12898, rs8898, and rs3779659 variants associated with PCOS risk	[273]
CTSD	Downregulated in ovaries of patients with PCOS	Cytoplasm and cell membrane of stromal and granulosa cells	Human	Abnormal follicle development	[271]
CTSS	Significantly increased levels in patients with PCOS	Serum	Human	Inflammation associated with PCOS	[272]

### 6.3. Primary Ovarian Insufficiency (POI)

Also known as premature ovarian failure (POF), POI is a condition characterized by the loss of ovarian function before the age of 40 [276,277]. The ovaries of mice with chemotherapy-induced POI showed increased expression of MMP2 and MMP9, and downregulation of TIMP-1 and TIMP-2 compared to control mice [278]. The greater activity of MMPs was associated with the increased apoptosis of the granulosa cells and abnormal folliculogenesis in the POI mice [278]. An et al. reported that the *TIMP2*G  >  C (rs8179090) and *TIMP2*G  >  A (rs2277698) genotypes were strongly associated with POI [279]. Pathogenic variants of serine protease *LONP1* have been found associated with POI [69]. Affected patients lack large antral follicles and are infertile, but the molecular mechanisms remain unknown, and functional analyses have not been performed [69]. PAs and PAIs are also associated with POI, particularly regarding the accelerated depletion of the ovarian reserve. Similarly to MMPs, tPA activity was higher in the ovaries of mice with chemotherapy-induced POI and associated with the increased apoptosis of granulosa cells and accelerated depletion of the ovarian reserve [280].

## 7. Conclusions

Proteases play crucial roles in several processes of ovarian function, including folliculogenesis, ovulation, and corpus luteum development. The balance between proteases and their inhibitors is essential for maintaining normal ovarian function. MMPs, PAs, and cathepsins contribute to the degradation of the follicular basement membrane and the extracellular matrix, enabling follicle activation, maturation, and oocyte release. The dysregulation of protease activity has been associated with several ovarian disorders, including cancer, PCOS, and POI. However, despite extensive evidence of the important role proteases play in ovarian functions, specific molecular mechanisms remain elusive. As proteases represent promising targets for therapeutic applications, future investigations should focus on identifying mechanisms of action and relationships with critical signaling pathways of the ovary.

## Abbreviations

The following abbreviations are used in this manuscript:

Abbreviation	Full Form
ADAMTS	A Disintegrin and Metalloproteinase with Thrombospondin-like Motifs
AIFM1	Apoptosis-Inducing Factor Mitochondrion-associated 1
AKT	Protein Kinase B
AMBP	Alpha-1-Microglobulin/Bikunin Precursor
ATG5	Autophagy Related 5
BCL2	B-Cell Lymphoma 2
CASP3/8	Caspase 3/8
CL	Corpus Luteum
COC	Cumulus–Oocyte Complex
CTSB	Cathepsin B
CTSD	Cathepsin D
CTSE	Cathepsin E
CTSG	Cathepsin G
CTSK	Cathepsin K
CTSL	Cathepsin L
CTSS	Cathepsin S
CstB	Cystatin B
Cst3	Cystatin C
CYP17A1	Cytochrome P450 Family 17 Subfamily A Member 1
CYP19A1	Cytochrome P450 Family 19 Subfamily A Member 1
DNA	Deoxyribonucleic Acid
Dpc	Days Post Coitus
Dpp	Days Postpartum
E2	Estradiol
ECM	Extracellular Matrix
EOC	Epithelial Ovarian Cancer
ERK	Extracellular Signal-Regulated Kinase
FAK	Focal Adhesion Kinase
FF	Follicular Fluid
FGF1	Fibroblast Growth Factor 1
FSH	Follicle-Stimulating Hormone
FURIN	Paired Basic Amino Acid Cleaving Enzyme
GCNA	Germ Cell Nuclear Antigen
GDF9	Growth Differentiation Factor 9
hCG	Human Chorionic Gonadotropin
HGF	Hepatocyte Growth Factor
ICSI	Intracytoplasmic Sperm Injection
IGF	Insulin-like Growth Factor
IGF1R	Insulin-like Growth Factor 1 Receptor
IL-1β	Interleukin 1 Beta
JNK	c-Jun N-terminal Kinase
KLK	Kallikrein
LC3-I	Microtubule-associated Protein 1A/1B-Light Chain 3
LH	Luteinizing Hormone
LONP1	Lon Peptidase 1
LMP	Low Malignant Potential
MAPK	Mitogen-Activated Protein Kinase
MMP	Matrix Metalloproteinase
mRNA	Messenger Ribonucleic Acid
mTOR	Mammalian Target of Rapamycin
MYC	Myelocytomatosis Oncogene
NFkB	Nuclear Factor Kappa B
P4	Progesterone
PA	Plasminogen Activator
PAI	Plasminogen Activator Inhibitor
PAPPA	Pregnancy-Associated Plasma Protein A
PAR1	Protease-Activated Receptor 1
PCNA	Proliferating Cell Nuclear Antigen
PCOS	Polycystic Ovary Syndrome
PI3K	Phosphoinositide 3-Kinase
PKC	Protein Kinase C
PMA	Phorbol-12-myristate 13-acetate
PMSG	Pregnant Mare Serum Gonadotropin
POF/POI	Premature Ovarian Failure/Primary Ovarian Insufficiency
PR	Progesterone Receptor
RGD	Arg-Gly-Asp (Arginine–Glycine–Aspartic acid)
ROS	Reactive Oxygen Species
SEC	Securities and Exchange Commission
siRNA	Small Interfering RNA
SMAD	Small Mothers Against Decapentaplegic
SPINK1	Serine Protease Inhibitor Kazal Type 1
STAR	Steroidogenic Acute Regulatory Protein
TGFα	Transforming Growth Factor Alpha
TGF-β	Transforming Growth Factor Beta
TIMP	Tissue Inhibitor of Metalloproteinases
TMPRSS6	Transmembrane Serine Protease 6 (Matriptase-2)
TNF-α	Tumor Necrosis Factor Alpha
tPA	Tissue-type Plasminogen Activator
TRAIL	TNF-Related Apoptosis-Inducing Ligand
TSRC1	Thrombospondin and Calcium-binding domains 1
uPA	Urokinase-type Plasminogen Activator
uPAR	Urokinase-type Plasminogen Activator Receptor
VEGF	Vascular Endothelial Growth Factor
VN	Vitronectin
ZMP-2	Zinc Metalloproteinase-2
ZP3	Zona Pellucida Glycoprotein 3
